# Cancer is chronic but antimicrobial stewardship is iconic: A retrospective cohort of optimal antibiotic use in ambulatory oncology clinics

**DOI:** 10.1017/ash.2023.152

**Published:** 2023-05-02

**Authors:** Tiffany A. Ho, Katelyn M. Patterson, Shirish M. Gadgeel, Rachel M. Kenney, Michael P. Veve

**Affiliations:** 1 Department of Pharmacy, Henry Ford Hospital, Detroit, Michigan; 2 Henry Ford Cancer Institute, Detroit, Michigan; 3 Division of Hematology and Oncology, Department of Internal Medicine, Henry Ford Hospital, Detroit, Michigan; 4 Department of Pharmacy Practice, Eugene Applebaum College of Pharmacy and Health Sciences, Wayne State University, Detroit, Michigan

## Abstract

**Objective:**

To evaluate antibiotic prescribing in ambulatory oncology clinics and to identify opportunities to improve antibiotic use.

**Methods:**

Retrospective cohort of adult patients who received care at 4 ambulatory oncology clinics from May 2021 to December 2021. Patients were included if they actively followed with a hematologist-oncologist for a cancer diagnosis and received an antibiotic prescription for uncomplicated upper respiratory tract infection (URTI), lower respiratory tract infection (LRTI), urinary tract infection (UTI), or acute bacterial skin–skin structure infection (ABSSSI) at an oncology clinic. The primary outcome was receipt of optimal antibiotic therapy, defined as a composite of drug, dose, and duration according to local and national guidelines. Patient characteristics were described and compared; predictors of optimal antibiotic use were identified using multivariable logistic regression.

**Results:**

In total, 200 patients were included in this study: 72 (36%) received optimal antibiotics and 128 (64%) received suboptimal antibiotics. The proportions of patients receiving optimal therapy by indication were ABSSSI (52%), UTI (35%), URTI (27%), and LRTI (15%). The most common suboptimal prescribing components were dose (54%), selection (53%) and duration (23%). After adjusting for female sex and LRTI, ABSSSI (adjusted odds ratio, 2.28; 95% confidence interval, 1.19–4.37) was associated with optimal antibiotic therapy. Antibiotic-associated adverse drug events occurred in 7 patients; 6 occurred patients who received prolonged durations and 1 occurred in a patient who received an optimal duration (*P* = .057).

**Conclusions:**

Suboptimal antibiotic prescribing in ambulatory oncology clinics is common and mostly driven by antibiotic selection and dosing. Duration of therapy may also be an area for improvement as national oncology guidelines have not adopted short-course therapy.

Advances in cancer therapeutics, testing, and antimicrobials with activity against multidrug-resistant organisms have allowed patients with cancer to live longer, but ∼60% of cancer-related deaths are due to infection.^1,2^ Antimicrobial stewardship is challenging in cancer patients due to uncertainties in infection diagnostics, complexities in immunocompromised population care, and the increased risk of infection and death due to multidrug-resistant organisms.^3^ Although contemporary cancer care has evolved from predominantly hospital-based practice to ambulatory settings (eg, care received in clinics, infusion centers, or via oral chemotherapies), the majority of antimicrobial stewardship efforts and practice remain in hospital settings.^4,5^ Ambulatory antimicrobial stewardship efforts are less mature and represent an area of needed growth, particularly in high-risk populations such as those with cancer.

Despite the high burden of infection, cancer patients are not well represented or are excluded from antimicrobial stewardship literature altogether, especially in ambulatory settings where antibiotic overuse is most common.^3,5,6^ In 2019, healthcare providers prescribed 251 million outpatient antibiotic prescriptions, and up to 50% were considered inappropriate.^7^ However, antibiotic prescribing practices in ambulatory oncology clinics are relatively unknown. This lack of data, challenges to prescriber education, fear of bad outcomes, just-in-case mentality, and the clinician perception of the patient demand for antibiotics are all drivers of suboptimal antibiotic use that are applicable to ambulatory oncology patients.^2,8,9^ Although more data are needed, current literature suggests that antimicrobial stewardship interventions developed and studied in general populations can be safely extrapolated to patients with cancer.^3,10^

Forming a baseline understanding of antibiotic prescribing practices and adherence to antimicrobial stewardship guidelines in the ambulatory oncology population is necessary to design and implement meaningful antimicrobial stewardship interventions.^5^ In this study, we evaluated antimicrobial prescribing in ambulatory oncology across a healthcare system that caters to a diverse patient population to identify areas to improve antibiotic use. We hypothesized that most antibiotics prescribed would be suboptimal, which would also lead to a high incidence of adverse events.

## Methods

### Study design

This retrospective cohort study was conducted across 4 outpatient cancer clinics within Henry Ford Health in southeastern Michigan. The study received institutional review board approval (no. 15246-01) with waiver of consent.

Patients were included in the study if they received an antibiotic prescription for an upper respiratory tract infection (URTI), lower respiratory tract infection (LRTI), urinary tract infection (UTI), or acute bacterial skin and skin structure infection (ABSSSI) from an ambulatory oncology clinic between May 1, 2021, and December 31, 2021. Additional inclusion criteria were age ≥18 years and active follow-up with a hematologist or oncologist for a cancer diagnosis at the time of care. Patients were excluded if they had multiple infections, were pregnant, were breastfeeding, were currently enrolled in a clinical trial, had been diagnosed with febrile neutropenia, or had had a previous treatment for infection of interest in the prior 30 days. Patients with a history of solid-organ or bone marrow transplant were excluded due to complexity in comparison to hematology-oncology populations and to prevent heterogeneity.

### Data source

Patient data were manually reviewed and extracted from the electronic health record (EHR) using the Epic Slicer Dicer data extraction tool (Epic Systems, Verona, WI). Data extraction parameters were set to identify patients who received an antibiotic prescription in an ambulatory oncology clinic during the prespecified timeframe. The identified patients were then randomized and screened for inclusion. Data collection included patient demographics, comorbid conditions including cancer diagnosis, infection characteristics (ie, risk factors for multidrug-resistant organisms), and antibiotic treatment. Patient outcomes included treatment failure, delayed chemotherapy, and adverse drug events. All data were captured by a single investigator using a standardized electronic case report form.

### Definitions

The primary end point of optimal antibiotic therapy was defined in accordance with the National Comprehensive Cancer Network (NCCN) guidelines and institutional antimicrobial stewardship guidelines was defined in accordance with the standard of care. Optimal treatment was defined as a composite of optimal drug, dose, and duration (Supplementary Table S1).^11^ The cohort was divided according to receipt of suboptimal or optimal treatment. Infection types were categorized according to medical record documentation; LRTI included chronic pulmonary obstructive disorder (COPD) exacerbation and community-acquired pneumonia (CAP); URTI included pharyngitis, bronchitis, and sinusitis; ABSSSI included purulent and nonpurulent cellulitis, abscess, and infected bite injuries; UTI included lower urinary tract infections (ie, cystitis in the absence of fever or flank pain suggestive of an upper UTI) and pyelonephritis. The full definitions of optimal regimens by disease state are listed in Supplementary Table 1. An analysis of prolonged antibiotic durations distinct from NCCN duration of therapy recommendations was performed and was defined as days of therapy exceeding institutional guideline recommended short-course durations (Supplementary Table S1).^12^

Secondary end points included the proportion of optimal components of the composite outcome, treatment failure, delay in chemotherapy due to active infection, and adverse drug events (ADEs) while on antibiotic therapy. We defined 30-day treatment failure as a documented change in antibiotic due to a lack of clinical improvement or due to a documented antibiotic adverse drug event, hospital admission due to worsening index infection, or development of recurrent infection. Only ADEs that were suspected to be due to the antibiotics the patient received were captured, as documented by the treating provider in the electronic health record (EHR).

All types of encounters including clinic visits, telephone encounters, and Epic MyChart EHR messages were reviewed for up to 30 days after antibiotics were initiated to find a documented secondary end point. Patients were presumed to be end point free at 30 days if they had no documented encounters.

### Statistical analysis

This study was designed to detect a 20% difference between any 2 variables associated with receipt of optimal antibiotics and assumed a baseline prevalence of 30% suboptimal antibiotic use. Assuming a type 1 error frequency of 5% and a power of 80%, a sample size of 200 patients was targeted. The assumption of 30% suboptimal antibiotic use was derived from prior internal data related to outpatient antibiotic prescribing at Henry Ford Health.^13^

Descriptive statistics (ie, proportion [%] and median [IQR]), were used to describe the cohorts of optimal and suboptimal antibiotic use. In bivariate analyses, categorical variables were compared using the Pearson χ^2^ or Fisher exact test, and continuous variables were compared using the Mann-Whitney *U* test. To determine variables independently associated with optimal antibiotic prescribing, variables associated with the outcome (*P* < .25) from bivariate analysis were entered into a multivariable regression model using a backward, stepwise approach. Variables included in the model were restricted to an event-to-variable ratio of 10:1; model fit was assessed using the Hosmer-Lemeshow goodness of fit test. A separate a priori analysis of optimal versus prolonged antibiotic duration of therapy was performed to assess associations with patient harm. All statistics were calculated using SPSS Statistics for Windows version 26.0 software (IBM, Armonk, NY).

## Results

In total, 200 patients were included in the study; 64% of patients received a suboptimal antibiotic course and 36% received an optimal antibiotic course. The most common infection types were UTI (41%), ABSSSI (31%), LRTI (17%), and URTI (11%). Cohort characteristics are listed in Table 1;we did not detect any statistical differences in patient-specific baseline characteristics between the 2 groups.

**Table 1.** Baseline Characteristics of Patients Receiving Suboptimal and Optimal Antibiotic Courses in Oncology Clinics

Variable	Total Population (*n=*200), No. (%)	Suboptimal Course (*n*=128), No. (%)	Optimal Course (*n*=72), No. (%)	*P* Value^a^
**Patient demographics**				
Age, median y (IQR)	68 (58–75)	69 (61–77)	66 (55–73)	.064
Sex, woman	130 (65)	77 (60)	53 (74)	.056
White race	118 (59)	77 (60)	41 (57)	.658
Public insurance	146 (73)	98 (77)	48 (67)	.130
β-lactam allergy	28 (14)	20 (16)	8 (11)	.377
History of MDRO within the prior 365 d	11 (6)	6 (5)	5 (7)	.53
Hospitalization within 90 d for >48 h	42 (21)	25 (20)	17 (24)	.497
Receipt of IV antibiotics within 90 d for >48 h	23 (12)	15 (12)	8 (11)	.897
Presence of CVC	112 (56)	77 (60)	35 (49)	.114
Face-to-face encounter	155 (78)	101 (79)	54 (75)	.525
**Cancer characteristics**				
Cancer diagnosis >1 year	99 (50)	63 (49)	36 (50)	.916
Solid tumor	167 (84)	105 (82)	62 (86)	.456
Hematological disease	33 (17)	23 (18)	10 (14)	.456
Actively receiving chemotherapy	112 (56)	71 (56)	41 (57)	.840
Curative intent of chemotherapy	38 (19)	25 (20)	13 (18)	.799
ECOG score >0	127 (64)	79 (62)	48 (67)	.485
ANC <100 cells/mm^3^	3 (2)	3 (2)	0 (0)	.371
Received GCSF during cycle of chemotherapy	13 (7)	10 (8)	3 (4)	.384
**Infection characteristics**				
Temperature >38°C (100.4°F)	5 (3)	2 (2)	3 (4)	.353
**Infection type**				
UTI	81 (41)	53 (41)	28 (39)	.73
ABSSSI	62 (31)	30 (23)	32 (44)	.002
LRTI	34 (17)	29 (23)	5 (7)	.005
URTI	22 (11)	16 (13)	6 (8)	.37

Note. ECOG, Eastern Cooperative Oncology Group; ANC, absolute neutrophil count; GCSF, granulocyte colony stimulating factor; MDRO, multidrug-resistant organism; IV, intravenous; CVC, central venous catheter; ABSSSI, acute bacterial skin and skin structure infection; UTI, urinary tract infection; LRTI, lower respiratory tract infection; URTI, upper respiratory tract infection.

a Refers to comparisons between patients who received suboptimal and optimal antibiotic courses.

**Fig. 1.**
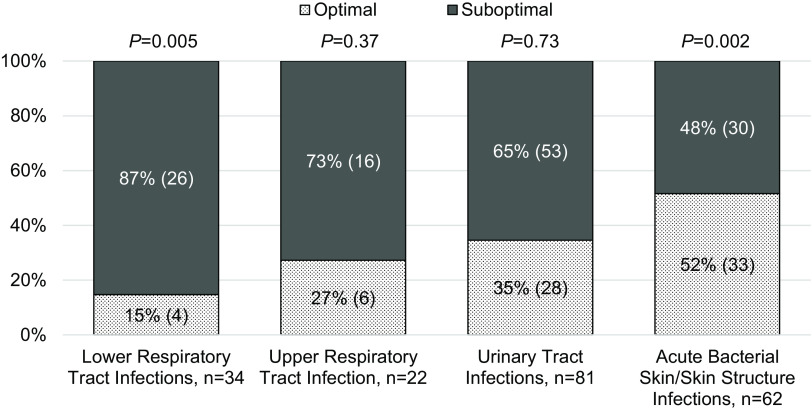
Optimal and suboptimal antibiotic use, by infection type, in outpatient oncology patients.

The proportion of optimal and suboptimal antibiotic use by indication is depicted in Figure 1. Optimal antibiotic use was most common in ABSSSI (52%) followed by UTI (35%), URTI (27%), and LRTI (15%). The most common component for optimal prescribing was duration (153, 77%). Optimal drug and dose occurred in 93 prescriptions (47%) and 92 prescriptions (46%), respectively.

Among patients with LRTI indications, CAP was notably associated with suboptimal antibiotic use due to selection of monotherapy instead of a CAP with comorbidities combination regimen or respiratory fluoroquinolone. Additional components of suboptimal antibiotic use by infection type are listed in Table 2.

**Table 2.** Suboptimal Antibiotic Use by Indication in 128 Outpatient Oncology Patients^a^

Characteristics (*n*, %)	LRTI (*n*=29)	URTI (*n*=16)	UTI (*n*=53)	ABSSSI (*n*=32)
Dose	25 (86)	15 (94)	36 (68)	31 (97)
Antibiotic selection	25 (86)	15 (94)	17 (32)	30 (94)
DURATION OF THERAPY	1 (3)	8 (50)	37 (70)	1 (3)
>1 suboptimal component	24 (83)	15 (94)	36 (68)	30 (94)

Note. LRTI, lower respiratory tract infection; URTI, upper respiratory tract infection; UTI, urinary tract infection; ABSSSI, acute bacterial skin and skin structure infection.

a Suboptimal was defined in accordance to a combination of institutional guidelines and NCCN recommendations. See Supplementary Table S1.^12^

Based on the results of the bivariate analyses and clinical rational, the following variables were included in a multivariable logistic regression model: female sex, public insurance, LRTI, ABSSSI, and presence of central venous catheter (Table 3). Other variables were excluded from the model due to unfit statistical criteria. ABSSSI was significantly associated with optimal prescribing (adjOR, 2.28; 95% CI, 1.19–4.37).

**Table 3.** Variables Associated With Receipt of Optimal Antibiotic Use in Outpatient Oncology Clinics^a^

Characteristic	Unadjusted OR (95% CI)	*P* Value	Adjusted OR (95% CI)	*P* Value
ABSSSI	2.54 (1.38–4.68)	.003	2.28 (1.19–4.37)	.003
Female sex	1.84 (0.982–3.48)	.056	1.85 (0.943–3.63)	.03
CVC	1.60 (0.892–2.86)	.197	Not tested	…
Public insurance	0.612 (0.323–1.16)	.130	Not tested	…
LRTI	0.231 (0.077–0.691)	.007	0.364 (0.116–1.16)	.143

Note. CI, confidence ratio; OR, odds ratio; ABSSSI, acute bacterial skin and skin structure infection; CVC, central venous catheter; LRTI, lower respiratory tract infection.

a Hosmer-Lemeshow test results: Methods: backwards logistic regression; variables removed from regression: presence of central venous catheter, public insurance; *P* = .659.

There was no statistically significant difference in the proportion of patients who experienced a 30-day treatment failure in the optimal and suboptimal antibiotic groups, respectively (15 [16%] vs 16 [15]; *P* = .819). Also, there was no statistically significant difference in the proportion of patients that had a delay in chemotherapy (4 [4%] vs 9 [8%]; *P* = .240), or any antibiotic-associated ADE (4 [4%] vs 3 [3%]; *P* = .707) between the optimal and suboptimal groups.

Of the 7 patients who developed an antibiotic-associated ADE, 3 developed an allergic reaction, 1 patient had diarrhea with macrolide use, 1 patient had acute kidney injury while receiving trimethoprim-sulfamethoxazole, 1 patient had QTc prolongation when receiving a fluoroquinolone, and 1 patient developed chills with arm rash and leg muscle spasms when receiving trimethoprim-sulfamethoxazole. Of 3 patients who developed an allergic reaction, 2 patients received a fluoroquinolone and 1 patient received trimethoprim-sulfamethoxazole.

### Associations with prolonged durations

Of 200 antibiotic regimens, 47% consisted of a prolonged duration that was defined as a duration outside the local guideline recommended short-course therapy and was distinct from more permissive NCCN recommendations. Variables associated with prolonged duration from bivariate analysis (*P* < .20) included non–face-to-face encounter (unadjusted odds ratio [unadjOR], 1.66; 95% confidence interval [CI], 0.85–3.3), female sex (unadjOR, 1.8; 95% CI, 0.99–3.3), ECOG score of 0 (unadjOR, 2.0; 95% CI, 1.1–3.6), history of infection or colonization with a multidrug-resistant organism within the previous year (unadjOR, 3.1; 95% CI, 0.79–11.9), history of IV antibiotic use within the previous 3 months (unadjOR, 1.8; 95% CI, 0.74–4.4), and ABSSSI (unadjOR, 5.7; 95% CI, 2.9–11.2). Outcomes associated with prolonged duration included recurrent infection within 30 days (unadjOR, 2.22; 95% CI, 0.89–5.5) and antibiotic-associated ADE (unadjOR, 6.9; 95% CI, 0.81–58.1).

## Discussion

In this study, 64% of patients had opportunities to improve the prescribed antibiotic regimen. The most common component of the regimen that was suboptimal was drug selection. A high percentage of optimal duration (77%) was expected because NCCN guidelines were utilized for the primary end point and had more lenient recommendations.^11^ However, in a secondary analysis of duration according to the institutional guidelines for short-course therapy, prolonged therapy was prescribed for 47% of patients. Existing literature demonstrates that short-course therapy is associated with fewer adverse drug events.^12,14^

Albeit limited, studies do exist that support stewardship efforts in immunosuppressed patients.^10^ The results of the present study are consistent with other literature that has evaluated antibiotic appropriateness in ambulatory oncology, in which prospective audit and feedback is not routinely performed.^15,16^ Future opportunities include use of EHR programming and decision support to optimize antibiotic selection, dose, and duration. Chew et al^15^ reported a low appropriateness of duration prescribed, which was attributed to the lack of a pre-set duration in the EHR. In response to these data, our institution implemented new EHR programming for pneumonia in patients with comorbidities, which prepopulates guideline-recommended regimens. Additional research is needed on short-course therapy. Existing randomized trials support short courses for febrile neutropenia.^17,18^ If shorter is better for the neutropenic host, we speculate that short-course therapy may also be safely applied to other oncology patients.

This study had several limitations. The retrospective study design was appropriate given the nature of the research question, but data collection was dependent on information documented within the EHR and vulnerable to patient loss to follow-up. However, with the ease of accessibility of providers through messaging or telephone, this information was captured if patients were inclined to notify their provider about ADEs. There was no application of strict definition for infection or colonization when considering optimal prescribing, and additional opportunities may exist to reduce unnecessary antibiotics for asymptomatic bacteriuria, bronchitis, and sinusitis. The external validity of study findings could be limited by our institution’s demographics and antibiotic prescribing patterns.

In conclusion, our findings suggest that opportunities for optimal antibiotic prescribing exist in ambulatory cancer care. Antimicrobial stewardship programs should leverage the EHR and existing clinical pharmacist infrastructure in ambulatory oncology clinics as a future direction for interventions to improve antibiotic prescribing and avoid patient harm. The design and implementation of meaningful and pragmatic antimicrobial stewardship interventions that promote appropriate short-course antibiotic therapy in ambulatory oncology clinics are an additional focus.

## Supplementary material

For supplementary material accompanying this paper visit http://doi.org/10.1017/ash.2023.152. click here to view supplementary material
