# Supplementary material for: Cancer is chronic but antimicrobial stewardship is iconic: A retrospective cohort of optimal antibiotic use in ambulatory oncology clinics
Source: Antimicrob Steward Healthc Epidemiol. 2023 May 2;3(1):e81. doi: 10.1017/ash.2023.152 (PMC10173287; doi:10.1017/ash.2023.152)
Supplement: Supplementary file 1 [file S2732494X23001523sup001.docx]

**Supplemental**

Table S1: Definitions of Optimal Antibiotic Treatment^†^

| **Infection** | | **Definition of Optimal Treatment** | **Guideline Used** | **Secondary Analysis of Short Course Therapy** |
| --- | --- | --- | --- | --- |
| **URTI** | Bronchitis | Empiric antibiotics are not recommended; symptom relief is suggested instead..  Zero days of antibiotic therapy are recommended. | - Institutional | Zero days |
|  | Sinusitis | ONLY consider treatment with antibiotics if patient meets criteria for acute BACTERIAL sinusitis which includes:   - Persistent signs or symptoms lasting ≥ 10 days without evidence of clinical improvement - Severe symptoms or signs of high fever and purulent nasal discharge or facial pain lasting for ≥ 3-4 consecutive days at beginning of illness - "Double sickening" with worsening symptoms or signs characterized by new onset of fever, headache, or increase in nasal discharge following typical viral upper respiratory infection that lasted 5-6 days and were initially improving   Treatment Recommendations for Acute Bacterial Sinusitis:   - Watchful waiting: encourage for uncomplicated sinusitis with reliable follow-up - Amoxicillin/clavulanate 875 mg/125 mg twice daily by mouth   Alternative Treatment for Patients with β-Lactam Allergy:  -Doxycycline 100 mg twice daily by mouth  Failure of Above Regimen or Suspected Resistance:  -Amoxicillin/clavulanate 2000 mg/125 mg XR twice daily by mouth  -Duration of 7-14 days | - Recommended duration per NCCN  - Recommended drug and dose per institutional guideline | 5 days |
|  | Pharyngitis | - Viruses are the most common cause of pharyngitis in all age groups. Antimicrobial therapy is indicated ONLY if Group A streptococci (GAS/streptococcus pyogenes) is confirmed in the pharynx by rapid antigen detection testing or culture in patients with SYMPTOMATIC pharyngitis.   Treatment for Group A Streptococci:  -Penicillin V (PO)500 mg by mouth twice daily for 10 days; drug of choice  -Amoxicillin 500 mg by mouth twice daily for 10 days  -Benzathine penicillin G   - < 27 kg: 600,000 units intramuscularly x 1 dose - > 27 kg: 1,200,000 units intramuscularly x 1 dose   Alternative Treatment for Group A Streptococci for Patients with β-Lactam Allergy:  -Cephalexin 500 mg by mouth twice daily x 10 days; avoid in individuals with immediate type hypersensitivity to penicillin.  -Azithromycin – 500 mg by mouth daily on day 1 and 250 mg by mouth daily on day 2-5 (use only for severe β-lactam allergy) | - Recommended drug, dose, duration per institutional guideline | Oral beta-lactam therapy – 10 days  Oral macrolide therapy – 5 days  Intramuscular therapy – 1 dose |
| **LRTI** | **CAP** | - Amoxicillin/clavulanate (2,000/125 mg by mouth twice daily or 875/125 mg by mouth three times daily) and azithromycin 500 mg daily - Amoxicillin/clavulanate (2,000/125 mg by mouth twice daily or 875/125 mg by mouth three times daily) and doxycycline 100 mg by mouth twice daily - Levofloxacin 750 mg by mouth daily - Moxifloxacin 400 mg by mouth daily - Duration of 5-14 days | - Recommended duration per NCCN  - Recommended drug and dose per institutional guideline | 5 days |
|  | **COPD Exacerbation** | -Doxycycline 100 mg by mouth twice daily for 5 days  Alternative: azithromycin 500 mg by mouth for 1 dose, then azithromycin 250 mg by mouth for 4 days. | - Recommended drug, dose, duration per institutional guideline | 5 days |
| **ABSSSI** | Non-purulent Cellulitis | - Cephalexin 500 mg to 1000 mg by mouth every 6 hours; drug of choice. Use 1000 mg dose in obese patients. - Dicloxacillin 500 mg by mouth every 6 hours - Clindamycin 300-450 mg by mouth three times daily; Reserve ONLY for patients unable to tolerate other therapies and monitor carefully for clinical response/failure. - Duration of 5-14 days | - Recommended duration per NCCN  - Recommended drug and dose per institutional guideline | 5 days |
|  | Purulent Cellulitis | Purulent Cellulitis is defined as cellulitis associated with an exudate or purulent drainage. Coverage for methicillin resistant *Staphylococcus aureus* infection is recommended.  -Trimethoprim/sulfamethoxazole DS 160/800 mg by mouth twice daily for 5 days  -Doxycycline 100 mg by mouth twice daily  -Clindamycin 300-450 mg by mouth three times daily. Reserve ONLY for patients unable to tolerate other therapies due to a high proportion of resistant *S. aureus* and monitor carefully for clinical response/failure.  -Duration of 5-14 days | - Recommended duration per NCCN  - Recommended drug and dose per institutional guideline | 5 days |
|  | Bites | Amoxicillin-clavulanate 875mg/125mg oral twice daily, or  Doxycycline 100mg oral twice daily or  Moxifloxacin 400 mg oral daily  Minimum of 7 days suggested; for pre-emptive therapy, 3-5 days of antibiotic therapy are recommended | - Recommended drug, dose, duration per institutional guideline | Bite – pre-emptive 3-5 days after cat or dog bite  Bite – 7 days after cat or dog bite |
| **UTI** | Cystitis | - Nitrofurantoin 100 mg by mouth for 5 days | - Recommended drug, dose, duration per institutional guideline | 5 days |
|  | Pyelonephritis | - Ciprofloxacin 500 mg twice daily for 7 days - Levofloxacin 750 mg once daily for 7 days   Consider alternatives if the patient has been exposed to a fluoroquinolone in the last three to six months   - Trimethoprim/sulfamethoxazole DS 160/800 mg twice daily for 7 days | - Recommended drug, dose, duration per institutional guideline | 7 days |

**Abbreviations:** NCCN, National Comprehensive Cancer Network; DS, double strength.

**†:**When considering dose for patients with renal impairment, institutional renal dosing guidelines were utilized to evaluate appropriateness.
